# No changes on viral load and CD4+ T-cell counts following immunization with 7-valent pneumococcal conjugate vaccine among HIV-infected adults in Malawi

**DOI:** 10.1016/j.vaccine.2018.04.009

**Published:** 2018-05-03

**Authors:** A.B. Ibarz-Pavon, N. French

**Affiliations:** a Centre for Global Vaccine Research, Institute of Infection & Global Health, University of Liverpool, United Kingdom; b Malawi-Liverpool-Wellcome Trust Laboratories, Blantyre, Malawi

**Keywords:** Viral load, CD4+ T-cell, PCV7, HIV-infected, Malawi

## Abstract

Vaccination has been associated with a transient increase in viremia in HIV-infected individuals, although contradicting evidence persist in the literature. As part of a randomized placebo-controlled efficacy trial of the PCV7 in Malawi, we collected viral load and CD4+ T-cell counts from 237 adults who received two doses of vaccine or placebo, administered 4 weeks apart. Analyses were conducted separately for cART and non-cART users. Our analysis show no difference in viral loads between vaccine and placebo groups, regardless of cART use. Viremia decreased from 4.1 to 2.9 log_10_ copies/mL (p < 0.0001) among those using cART, consistent vaccine and placebo groups, but no changes were seen among the non-cART cohort. CD4+ T-cell counts remained unchanged regardless of cART use, or allocation to vaccine or placebo. We concluded that there was no evidence of detrimental effects of PCV7 administration on viral load or CD4+ T-cell counts six months after vaccination with PCV7.

## 1 Introduction

*Streptococcus pneumoniae* remains one of the biggest health threats in sub-Saharan Africa and, particularly, to the HIV infected population [1], but can be partly prevented with pneumococcal conjugate vaccines (PCV) [2], which have been effective in reducing the burden of disease among both HIV-infected and non-infected individuals, as well as providing indirect protection among those unvaccinated [2], [3], [4], [5], and are recommended by policy-making bodies [6], [7], [8]. Immunization is considered safe, but several vaccines recommended for HIV+ patients have been linked to short-term increases in plasma viral loads (VL) [9], [10], [11], although this finding is inconsistent [12], [13], [14], [15]. Immunization of HIV+ patients with pneumococcal polysaccharide vaccine was associated with transient increased viremia in a study conducted before combination Antiretroviral Therapy (cART) was available [16], but subsequent studies failed to corroborate this finding [14], [17], [18]. Three studies measured the impact of PCV and found no evidence of increases on VL, independently of cART [3], [4], [14]. Here, we analysed data collected during a double-blind randomized placebo-controlled trial evaluating the efficacy of the PCV7 among Malawian adults [2]. A *post-hoc* analysis of that study showed an excess mortality among the HIV+ PCV7-vaccinated subgroup who did not receive cART. We hypothesised that PCV7 might have a detrimental effect on HIV control, and report a comparison of changes in VL and CD4+ T-cell counts post-vaccine between two groups: those who received cART, and those who did not. cART use in our cohort was primarily determined by date of recruitment, with public funded cART becoming available two years after recruitment started.

## 2 Methods

The methods of this study are described elsewhere [2]. Briefly, individuals were enrolled following an episode of invasive pneumococcal disease. They received two doses of either PCV7 or a placebo, administered four weeks apart. Blood samples were collected at enrolment, before vaccination, and six months later. A total of 496 patients were recruited for the original trial, of which 439 (88.5%) were HIV+. Of these, 220 received the PCV7, and 219 a placebo. There were 136 (31.0%) deaths, 64 (47.1%) of which occurred within 6 months of recruitment, and 55 (86%) of these occurred among non-cART receivers. Sixty-seven (14.6%) HIV+ patients were lost to follow-up, of which 30 (44.8%) were lost within 6 months, of which 27 (90%) were on the non-cART group. The study was approved by the College of Medicine Research and Ethics committee (protocol number 99/00/101).

For this analysis, we included 237 HIV+ cases for whom VL data at enrolment and at 6-months follow-up were available (Table 1). Analyses were conducted separately for individuals receiving cART, defined by already being on cART at enrolment or commnencing it at any point between the baseline and follow-up sample being taken, and those who did not receive cART at any point in-between samplings. Normally-distributed variables are expressed as mean and standard deviation, and non-normally distributed ones are defined by median and interquartile range. Categorical variables were compared using Pearson’s Chi-squared or Fisher’s exact test. Student’s *t*-test, and Wilcoxon Rank-Sum test were used for continuous variables. VLs and CD4+ T-cell counts were log_10_-transformed to approximate a normal distribution, and mean values between baseline (t0) and at 6-months (t6), and between and within vaccine and placebo groups were compared using a two-tailed paired and unpaired Student’s *t*-test respectively. Statistical significance was set at p < 0.05. Analyses were conducted with StataV.12.1 (STATA Corp, TX, USA).

**Table 1:** Population characteristics and changes in CD4+ T-cells and Viral Loads pre-and post-intervention.

	All population	ON cART (n = 79)			NOT ON cART (n = 158)		
		Vaccine (n = 38)	Placebo (n = 41)	p value	Vaccine (n = 83)	Placebo (n = 75)	p value
**Patients included n (%)**	237	38 (16.0)	41 (17.3)		83 (35.0)	75 (31.6)	
Age, mean (SD)	34.4 (9.7)	36.9 (7.8)	35.5 (10.1)	0.754	33.3 (10.7)	33.8 (9.1)	0.380
Male gender, n (%)	95 (40.1)	8 (21.1)	13 (31.7)	0.284	42 (50.6)	32 (42.7)	0.318
**Total receiving cART at any point between samplings**	79 (33.3)	38	41		NA	NA	
On cART at enrolment, n (%)	32 (40.5)	17 (44.7)	15 (36.6)		NA	NA	
Started cART within 6 months of enrolment, n (%)	47 (59.5)	21 (55.3)	26 (63.4)		NA	NA	

*VL and CD4 COUNTS AT ENROLMENT (t0)*							
**CD4, median (range), cells/µL**	192 (7–9380)	181 (32–573)	191 (23–626)	0.810	245 (40–835)	183 (7–938)	0.194
Missing data, n (%)	22 (9.28)	3 (7.9)	6 (14.6)		8 (9.6)	5 (6.7)	
**Viral load, mean (SD), log10 copies/mL**	4.6 (1.1)	4.0 (1.3)	4.2 (1.3)	0.308	4.7 (0.8)	4.9 (0.8)	0.100
VL > 5log10 copies/mL, n (%)	107 (45.15)	16 (42.1)	15 (36.6)	0.616	39 (47.0)	37 (49.3)	0.768
Undetectable VL (<400 copies/mL), n (%)	35 (14.8)	16 (42.1)	14 (34.2)	0.495	4 (4.8)	1 (1.3)	0.370

*VL and CD4 COUNTS AT 6 MONTHS (t6)*							
**CD4, median (range), cells/µL**	229 (3–2000)	287 (81–770)	247 (23–616)	0.332	238 (8–2000)	199 (3–824)	0.009
Missing data, n (%)	24 (10.1)	4 (10.5)	1 (2.4)		11 (13.3)	8 (10.7)	
**Viral load, mean (SD), log10 copies/mL**	4.2 (1.2)	2.8 (0.7)	3.0 (0.9)	0.274	4.8 (0.7)	4.9 (0.7)	0.121
VL > 5log10 copies/mL, n (%)	90 (38.0)	2 (5.3)	3 (7.3)	0.708	42 (50.6)	43 (57.3)	0.397
Undetectable VL (<400 copies/mL), n (%)	65 (27.4)	31 (81.6)	31 (75.6)	0.591	2 (2.4)	1 (1.3)	1.00

## 3 Results

Of the 439 HIV+ individuals recruited for the original clinical efficacy trial, 237 provided both t0 and t6 blood samples. Seventy-nine (33.3%) were on cART at some point between samplings, of which 32 (40.5%) were on cART at enrolment: 17 on the vaccine and 15 on the placebo arm, and 47 (21 in vaccine and 26 in placebo) started within 6 months of recruitment (Table 1). The mean times to cART commencement among these were 108.6 ± 62.1 and 93 ± 52.9 days for placebo and vaccine arms respectively (p = 0.365).

For both, cART and non-cART subgroups, participants in the vaccine and placebo arms were similar in regard of age, gender, and t0 VL and CD4+ T-cell counts. The mean time between t0 and t6 sampling was 184.5 ± 9.2 days, with no differences in regard of cART use (184.7 ± 8.8 on cART users *vs.* 184.3 ± 9.4 non-cART), and vaccine or placebo group (184.8 ± 9.2 and 184.2 ± 9.2 respectively).

There were no significant differences on VL baseline values between vaccine and placebo groups at t0 nor at t6 in either those receiving cART (t0 p = 0.308, t6 p = 0.274), nor on those not on cART within the sampling period (t0 = 0.100; t6 p = 0.121). Among those receiving cART, we observed a reduction on VL between t0 and t6, from 4.1 to 2.9 log_10_ copies/mL (diff 1.2 [0.89–1.51] p < 0.0001). which was consistent in vaccine (diff 1.18 [0.73–1.62] p < 0.0001 and placebo diff 1.22 [0.79–1.65] p < 0.0001) groups. This reduction was not observed among those not on cART.

Among the cART group, the number of individuals with undetectable VL (VL < 400 copies/ml) increased from 16 (42.1%) at t0 to 31 (81.6%) at t6 among vaccine recipients, and from 14 (34.2%) to 31 (75.6%) in the placebo. Among the non-cART group, 4 (4.8%) and 2(2.4%) vaccine recipients had undetected VLs at t0 and t6 respectively. One patient presented a VL < 400 cell/mL at both sampling points in the placebo group.

CD4+ T-cell counts were no different between vaccine and placebo groups at t0 and t6 regardless of cART treatment subgroup. Those receiving cART had an average increase of 55.7 CD4+ T-cell counts at t6 compared to t0 (p < 0.0001), which was consistent in vaccine (diff 60.6 p = 0.0004) and placebo (51.4 p = 0.0042) arms. No significant changes were observed between groups among those not on cART.

## 4 Discussion

Results show no evidence of a PCV7-induced change on either VL or CD4+ T-cell cell counts in HIV+ adults six months after receiving two doses of PCV7, consistent with published studies [3], [4], [14], which reassures us of our findings despite the fact that the sample size in all subgroups is relatively small and could have masked the effects. The reduction on VL detected among those receiving cART could be explained by use of cART, and was comparable in vaccine and placebo groups. We undertook this analysis looking for evidence of VL resetting resulting from vaccination that might provide an explanation for the observed increased mortality among those on the non-cART vaccine group in the original study [2], and found no evidence. We cannot exclude earlier changes in VL that other reports have suggested [11], [19], but are reassured that there are no long-term detrimental effects on HIV infection or its control among HIV+ patients receiving cART. We cannot rule out the possibility that vaccine-induced increases in VL within the first 6 months of enrolment, which we did not measure, might have contributed to early deaths. However, in our population, these individuals presented a lower baseline CD4+ T-cell counts (117 cells/µL [2–753]), and higher VLs (4.9 log_10_copies/mL) than those who survived, consistent with a higher risk of death independent of vaccine administration. Those who were loss to follow-up within 6 months could not be included in our analysis, as they would not have provided the second sample. The majority of these subjects were not receiving cART, and while their baseline VL and CD4+ T-cell values were not significantly different from those who remained in the study (data not shown), we cannot rule out an effect that could pretentially skew the comparison.

This study shows that the administration of two doses of PCV7 to HIV+ individuals does not have a detrimental effect on VL or CD4+ T-cell counts in HIV+ individuals, regardless of whether they receive cART therapy. Based on these findings, the initial hypothesis of the excess deaths on the vaccine group observed in the original study being the result of attrition due to higher loss to follow-up among the placebo remains a plausible explanation [2].
